# Gastrointestinal Parasites of Domestic Mammalian Hosts in Southeastern Iran

**DOI:** 10.3390/vetsci10040261

**Published:** 2023-03-29

**Authors:** Kareem Hatam-Nahavandi, David Carmena, Mostafa Rezaeian, Hamed Mirjalali, Hanieh Mohammad Rahimi, Milad Badri, Aida Vafae Eslahi, Farzaneh Faraji Shahrivar, Sonia M. Rodrigues Oliveira, Maria de Lourdes Pereira, Ehsan Ahmadpour

**Affiliations:** 1 Department of Parasitology and Mycology, School of Medicine, Iranshahr University of Medical Sciences, Iranshahr 9916643535, Iran; 2 Parasitology Reference and Research Laboratory, Spanish National Centre for Microbiology, Health Institute Carlos III, 28220 Majadahonda, Spain; 3 CIBERINFEC, ISCIII—CIBER Infectious Diseases, Health Institute Carlos III, 28029 Madrid, Spain; 4 Department of Parasitology and Mycology, School of Public Health, Tehran University of Medical Sciences, Tehran 1416634793, Iran; 5 Foodborne and Waterborne Diseases Research Center, Shahid Beheshti University of Medical Sciences, Tehran 1985714711, Iran; 6 Medical Microbiology Research Center, Qazvin University of Medical Sciences, Qazvin 3419915315, Iran; 7 Department of Physiology, School of Medicine, Iranshahr University of Medical Sciences, Iranshahr 9916643535, Iran; 8 CICECO—Aveiro Institute of Materials, University of Aveiro, 3810-193 Aveiro, Portugal; 9 Hunter Medical Research Institute, New Lambton, NSW 2305, Australia; 10 Department of Medical Sciences, University of Aveiro, 3810-193 Aveiro, Portugal; 11 Drug Applied Research Center, Tabriz University of Medical Sciences, Tabriz 5166614766, Iran

**Keywords:** cattle, camel, donkey, sheep, goat, dog, *Giardia duodenalis*, *Eimeria* spp., *Entamoeba* spp., *Trichuris* spp.

## Abstract

**Simple Summary:**

Parasitosis of the digestive tract by worms and protozoa in livestock is a veterinary health and economic concern as parasitic infections can cause deterioration of animal welfare and reduced productivity associated with delayed growth rate, weight loss, and reduced milk production. Some parasites of the digestive tract have zoonotic potential, so farm animals can act as source of human infections. This study investigated the prevalence of intestinal parasites in cattle, camels, donkeys, horse, sheep, goats, and dogs from Iranshahr County in Southeastern Iran. Our findings indicate that most animals studied were infected with at least one species of intestinal parasite. Parasitological monitoring, including testing during the rearing of free-range animals, is needed in livestock to detect carriers and shedders of parasite eggs, cysts, and oocysts. It is also recommended that villagers prevent stray dogs from entering agricultural fields and ensure the proper housing and management of animal’s facilities, with special attention to their hygienic conditions. Furthermore, accurate diagnosis of parasitic infections, as well as effective monitoring and prophylaxis, are essential to keep livestock free of parasitic infections.

**Abstract:**

Gastrointestinal parasites (GIP) are a major cause of disease and production loss in livestock. Some have zoonotic potential, so production animals can be a source of human infections. We describe the prevalence of GIP in domestic mammals in Southeastern Iran. Fresh fecal samples (*n* = 200) collected from cattle (*n* = 88), sheep (*n* = 50), goats (*n* = 23), camels (*n* = 30), donkeys (*n* = 5), horse (*n* = 1), and dogs (*n* = 3) were subjected to conventional coprological examination for the detection of protozoan (oo)cysts and helminth ova. Overall, 83% (166/200) of the samples were positive for one or more GIP. Helminths were found in dogs, donkeys, sheep (42%), camels (37%), goats (30%), and cattle (19%), but not in the horse. Protozoa were found in cattle (82%), goats (78%), sheep (60%), and camels (13%), but not in donkeys, dogs, or the horse. Lambs were 3.5 times more likely to be infected by protozoa than sheep (OR = 3.5, 95% CI: 1.05–11.66), whereas sheep were at higher odds of being infected by helminths than lambs (OR = 4.09, 95% CI: 1.06–16.59). This is the first study assessing the prevalence of GIP in domestic mammals in Southeastern Iran.

## 1. Introduction

Gastrointestinal parasites (GIP) are major threats to animal health, often causing considerable economic losses in production animals associated to reduced growth and reproduction rates, lower production of milk/meat and, very often, death [1]. In tropical and subtropical areas, GIP are one of the major menaces for livestock, causing constraints to the development of a profitable livestock industry [2,3,4,5,6]. Investigating the disease burden of GIP in domestic animals is, therefore, an important aspect for animal welfare and management [7]. Furthermore, since domestic mammals can act as suitable reservoirs for zoonotic parasites, they may serve as disregarded sources of human parasitic infections [8].

Protozoan parasites of the genera *Entamoeba* and *Giardia* are globally distributed pathogens (some of them with zoonotic potential) that infect humans and a wide diversity of domestic (e.g., cattle, sheep, and goats) and wild (e.g., non-human primates) animal species, imposing a significant threat on human and veterinary health in endemic areas [9,10,11,12]. In addition, livestock may have an important (although not fully clarified) role in the transmission of zoonotic diseases to humans via direct contact with animal manure or indirectly through ingestion of contaminated food or water [13,14,15]. Stray dogs are reservoirs and carriers of several zoonotic intestinal parasites that are considered serious problems for human, such as canine echinococcosis [16,17].

In Southeastern Iran, the risk of zoonotic transmission of some GIP via domestic mammals should be carefully considered because most livestock are reared as free-range animals with open access to agriculture fields and surface water sources. Such a rearing system increases environmental contamination with parasitic transmission stages (e.g., cysts, oocysts), facilitates contact between domestic animals and humans, and favors the spreading of parasitic pathogens to new hosts and environments. Stray dogs roam freely in these farmlands. Each contact between the suitable new host and infected feces in the environment represents a potential parasite transmission event [18]. Hence, the present study aimed at determining the prevalence and risk factors of GIP in cattle, camels, donkeys, a horse, sheep, goats, and dogs from across Iranshahr County in Southeastern Iran. These findings were compared with previous findings on GIP in the surveyed host species in Iran and elsewhere.

## 2. Materials and Methods

### 2.1. Study Area

**Figure 1:**
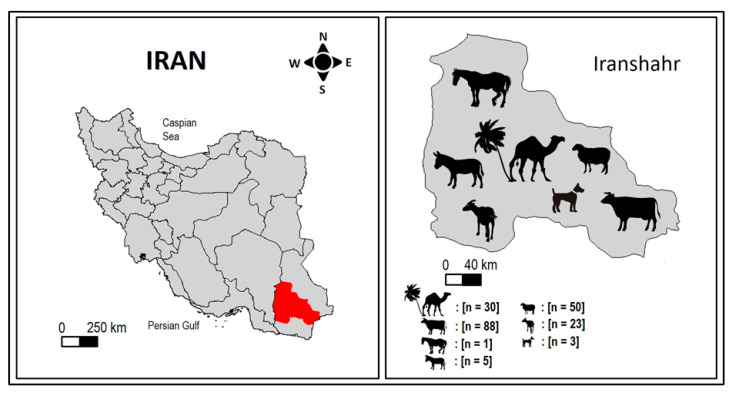
Map of Iran showing the geographical location of Iranshahr County (left-hand side of the figure) where the sampling of different domestic animal species (right-hand side of the figure) was conducted.

This study was carried out in Iranshahr County (Figure 1), in the middle of the Sistan and Baluchestan province (27°12′9″ N, 60°41′5″ E) in Southeastern Iran. The region covers an extent of about 4,173,000 ha at an average height of 591 m above sea level. It has a dry, hot, and windy climate, a mean annual temperature of 32 °C, and an average annual rainfall of 114.7 mm. Precipitation, scarce and falling mainly in heavy rainstorms, causes severe flooding, while the heat is excruciating for eight months of the year.

The Iranshahr County is characterized by a low density of domestic animals. In the traditional farming system, cattle are free ranging in hay fields surrounded by palm trees. Each family of local villagers typically rears 1–5 cattle semi-intensively in backyards with soil-type flooring. Animals are routinely released for grazing during daytime and sheltered at night in partially covered small buildings.

### 2.2. Sample Size Estimation

According to the formula (*n* = Z2 P (1 − P)/d2) [19] [where *n* = the required sample size, Z = 95% confidence level (1.96), P = expected prevalence (80–100% in camels, cattle, goats and sheep) [12,20,21], and d = precision (0.09)] the minimum sample size required for this study was estimated at ~40 for each animal species considered. It should be noted that animals such as horses and donkeys are rarely found in the southeastern regions of Iran. Therefore, fecal samples were collected opportunistically from these animals when we found them by chance in crossings close to agricultural fields.

### 2.3. Sample Collection

Two hundred fresh fecal samples were individually collected from different domestic animal species including cattle (*Bos taurus* and *Bos indicus*, *n* = 88), sheep (*Ovis aries*, *n* = 55), Arabian camels (*Camelus dromedarius*, *n* = 30), goats (*Capra hircus*, *n* = 23), donkeys (*Equus asinus*, *n* = 5), horses (*Equus caballus*, *n* = 1), and stray dogs (*Canis lupus familiaris*, *n* = 3). Animals were aged between 2 months and 10 years and were asymptomatic at the moment of sampling. The study was conducted at regular intervals during May and September 2022. Fecal samples were taken directly from the rectum of ruminant animals with sterile plastic gloves and placed into 50 mL conical-bottom tubes. Freshly voided fecal samples of canine origin were directly collected from the ground. Fecal samples were excluded from the study if they were collected from the ground and could not be associated with a specific host species. A single fecal sample was collected from each investigated animal (200 samples vs. 200 animal). Each fecal sample was assigned to a unique identification code. Information regarding sampling date, origin, host species, age, and sex was recorded. The collected samples were transported in cooled boxes to the Parasitology Research Laboratory of Iranshahr University of Medical Sciences (IRSHUMS) and stored at 4 °C until further processing.

### 2.4. Coprological Analysis

The modified Ritchie’s formol-ether sedimentation technique was used to concentrate (oo)cysts, ova, and larvae present in the fecal samples collected [22]. Briefly, 10 g of fecal material per sample was mixed with 50 mL of tap water in a disposable plastic cup; this mixture was poured into an Erlenmeyer flask and enough water added to make a total volume of 300 mL; 15 mL was then poured, without filtering, directly into a 15 mL graduated conical centrifuge tube and centrifuged at 500× *g* for 2 min. After removing the supernatant, 12 mL of water were added to resuspend the sediment. Then, 3 mL of diethyl ether was added, and the homogenate was centrifuged at 300× *g* for 2 min. The resulting layer of diethyl ether containing debris and water were removed. The remaining sediment was diluted in a few drops of distilled water and 20 µL were placed onto a clean glass slide and a coverslip was placed on the mixture. The slide was subsequently examined under light microscopy at 100× and 400× magnification (Nikon, ECLIPSE E100, Tokyo, Japan). Fecal samples that were microscopically positive for *Eimeria* were incubated in 2.5% (*w*/*v*) potassium dichromate solution at 25–28 °C for 15 days to sporulate the oocysts of the parasite [23]. Morphological identification of GIP was conducted according to standard keys [24].

### 2.5. Statistical Analysis

Epidemiological and diagnostic data generated in the present study were entered into a Microsoft Office Excel 2016 spreadsheet and exported to the IBM SPSS^®^ Statistics package, version 25 (IBM Corp., Armonk, NY, USA) for statistical analysis. The baseline characteristics of the studied animals were summarized using frequencies into categorical variables. Binomial logistic regression was used to analyze the strength of the association between dependent (infection [positive vs. negative]) and dichotomous independent variables (sex [male vs. female] and age [≤1 yr. vs. >1 yr.]). Probability values lower than 0.05 were considered statistically significant.

## 3. Results

### 3.1. Parasitological Findings

Overall, 83.0% (166 of 200) of the fecal samples screened were positive at microscopy examination for one or more GI parasite (Table 1). Single infections were detected in 83.0% (166/200) and co-infections by two or more GIP species in 39.5% (79/200). Protozoan (oo)cysts and helminth ova were detected in 61.0% (122/200) and 31.0% (62/200) of fecal samples, respectively. By host species, dogs had the highest infection rate by any given GIP (100%, 3/3) followed by sheep (94%, 47/50), cattle (87.5%, 77/88), goats (86.9%, 20/23), donkeys (80%, 4/5), and camels (46.6%, 14/30).

Helminthic infections were more frequent in dogs (100%, 3/3) followed by donkeys (80.0%, 4/5), sheep (42.0%, 21/50), camels (36.6%, 11/30), goats (30.4%, 7/23), and cattle (19.3%, 17/88). No helminthic infections were detected in the only horse sample available in the study (Table 1). Nematodes accounted for most (28.5%, 57/200) of the helminthic infections detected, followed by cestodes (2.0%, 4/200) and trematodes (1.0%, 2/200).

Protozoan infections were more frequent in cattle (81.8%, 72/88), followed by goats (78.2%, 18/23), sheep (60.0%, 30/50), and camels (13.3%, 4/30). No protozoan infections were detected in dogs, donkeys, and the horse. *Entamoeba* spp. accounted for most (34.0%, 68/200) of the protozoan infections detected.

**Figure 2:**
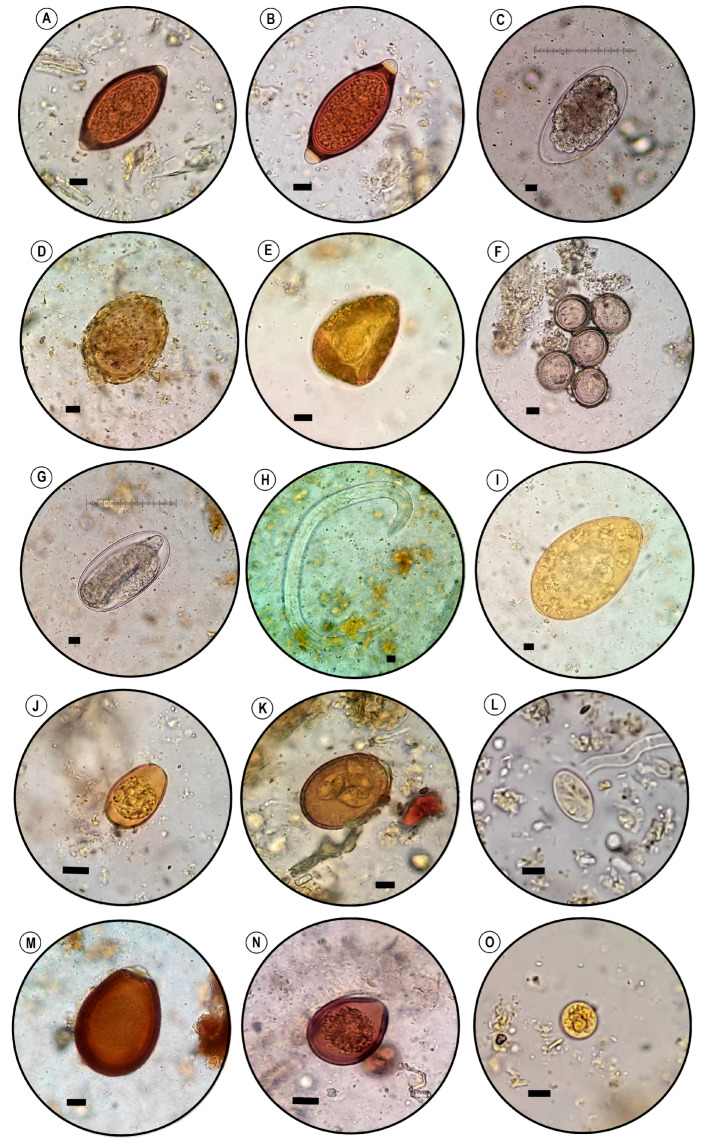
Images of GIP in fecal samples of domestic animals observed in the present study. (**A**) *Trichuris cameli* ova (camel). (**B**) *Trichuris ovis* ova (sheep). (**C**) Trichostrongyle-type ova (cattle). (**D**) Ascarid-like pollen grain (cattle). (**E**) *Moniezia expansa* (camel). (**F**) *Taenia* spp. ova (dog). (**G**) Embryonated Strongyle-type ova (donkey). (**H**) Rhabditiform larva (cattle). (**I**) Amphistome ova (cattle). (**J**) *Eimeria auburnensis* unsporulated oocyst (cattle). (**K**) *Eimeria intricata* sporulated oocyst (sheep). (**L**) *Giardia duodenalis* cyst (sheep). (**M**) *Eimeria cameli* unsporulated oocyst (camel). (**N**) *Eimeria bukidnonensis* unsporulated oocyst (cattle). (**O**) *Entamoeba* spp. cyst (cattle). Scale bar: 10 µm.

Overall, nine types of GIP species were identified. These included the protozoa *Entamoeba* spp., *Eimeria* spp., and *Giardia duodenalis*. Among cestodes, we detected two types of cyclophillidean (*Taenia* spp., *Moniezia expansa*), a single digenean (amphistomes the order Echinostomida), and three types of nematodes including strongyles, trichostrongyles, and Trichuridae (Table 1 and Figure 2).

Table 2 shows the GIP infection rates according to host species. Among protozoa, *Eimeria* infections were frequent in domestic ruminant species, particularly in ovine and caprine animals. Thus, 82.6% (19/23, 95% CI: 61.2–95.1) of goats harbored *Eimeria* oocysts belonging to *E. arloingi* (55.5%, 10/18), *E. ninakohlyakimovae* (33.3%, 6/18), and *E. christenseni* (16.6%, 3/18). Co-infection of *E. arloingi* and *E. ninakohlyakimovae* was identified in one case (5.5%). A lower prevalence 52.0% (26/50; 95% CI: 37.4–66.3) was found in sheep, which were infected by three distinct *Eimeria* species including *E. ahsata* (57.7%, 15/26), *E. crandallis* (38.5%, 10/26), and *E. intricata* (3.8%, 1/26). *Eimeria* infection rate in cattle was 13.6% (12/88, 95% CI: 7.2–22.6), with three species being identified in this host: *E. auburnensis* (66.6%, 8/12), *E. ellipsoidalis* (25%, 3/12), and *E. bukidnonensis* (8.3%, 1/12). A very similar figure was identified in camels (13.3%, 4/30; CI: 3.7–30.7), which were infected by host-adapted species including *E. cameli* (75.0%, 3/4) followed by *E. dromedarii* (25.0%, 1/4) (Table 1).

*G. duodenalis* cysts were found in 4.0% of sheep (2/50, 95% CI: 0.5–13.7). Both infected animals were male, one <1 yr. and the other >1 yr. *G. duodenalis* cysts were also detected in 4.3% of goats (1/23, 95% CI: 0.1–21.9). The infected animal was a 3-months old male kid. *Entamoeba* spp. was a frequent finding in cattle (72.8%, 64/88; 95% CI: 62.2–81.7). Comparatively, lower prevalence rates were identified in sheep (6.0%, 3/50; 95% CI: 1.2–16.5) and goats (4.3%, 1/23; 95% CI: 0.1–21.9) (Table 1).

The most prevalent helminth species in cattle were Trichostrongyle spp. (family Trichostrongylidae; 82.3%, 14/17), followed by *Trichuris* spp. (family Trichuridae; 5.8%; 1/17) and Amphistome spp. (family Paramphistomatidae; 11.7%, 2/17). Trichostrongyle spp. (85.7%, 18/21) were also the most abundant helminth parasites found in sheep, followed by *Trichuris* spp. (14.3%, 3/ 21). Similar results were found in goats, infected by Trichostrongyle spp. (57.2%, 4/7) and *Trichuris* spp. (42.8%, 3/7). We found three helminth species infecting camels, being the most prevalent Trichostrongyle spp. (63.6%, 7/11), followed by *Trichuris* spp. (27.3%, 3/11), and *M. expansa* (family Anoplocephalidae, 9.1%, 1/11). Finally, strongyle spp. (family Strongylidae) was found in 80% (4/5) of donkeys and *Taenia* spp. (family Taeniidae) in 100% (3/3) of dogs. In the latter host, motile proglottids (12 × 6 mm), similar to those of *T. multiceps* and *T. hydatigena*, were observed at macroscopic examinationof the fecal samples. Liver flukes of the genera *Fasciola* and *Dicrocoelium* were not detected in any of the fecal samples from ruminants examined.

### 3.2. Risk Analysis

Sheep ≤ 1 year were more likely to harbor infections by protozoan parasites than their older counterparts (75.0% vs. 46.2%); this difference was statistically significant (χ2 = 4.33, *p* ≤ 0.037). In contrast, adult sheep were at higher risk of being infected by helminth species than young sheep (57.7% vs. 25.0%); this difference was also statistically significant (χ2 = 5.48, *p* ≤ 0.019).

Univariate analyses confirmed that age was a determining factor in the occurrence of protozoan and helminth infections in the surveyed sheep population, with young (≤1 yr.) animals being at higher odds of having a protozoasis (350%) and old (>1 yr.) animals of having helminthiasis (409%) (Table 3).

## 4. Discussion

The main contribution of this study is the demonstration that GIP infections are very common (83%) in livestock in Southeastern Iran, with protozoasis (61%) being more prevalent than helminthiasis (31%). Similarly high infection rates have also been documented in a previous study conducted in Poland, where 87.8% and 79.2% of goats in conventional and organic farms, respectively, were infected with GIPs [25]. Likewise, previous research from India has reported that 85.1% and 79.2% of sheep and goats, respectively, were infected with GIPs [20]. As GIPs are mainly transmitted through the fecal–oral route, the high infection rates identified in the present study can be explained by the conditions of raising and management of the investigated animal populations. Livestock were reared as free-range animals with open access to agriculture fields and surface water sources. Therefore, fecal contamination of grassland and water by infected livestock may facilitate the occurrence and frequency of reinfection events. There may be a strong ecological interdependence between the spread of GIPs and the migration of nomadic groups in these areas. Many Baloch tribes in the Baluchestan region between the three countries of Iran, Pakistan, and Afghanistan, moved many herds of livestock during the extensive seasonal movements, and certainly these movements have transported many GIPs in these areas. On the other hand, large-scale animal smuggling without any veterinary supervision and livestock quarantine requirements causes the spread of many parasites across borders in these areas, which makes their control difficult. In the same way, many smugglers load large amounts of opium onto camels, which move without a camel driver and reach their destination outside the borders of Afghanistan, causing the spread of GIPs in these areas.

In the present study, the most prevalent protozoa were uninucleated *Entamoeba* spp., which were primarily found colonizing/infecting cattle (72.8%), and, to a much lesser extent, sheep (6.0%) and goats (4.3%). These results were in accordance with those found in previous studies on livestock populations in African countries including cattle in Uganda (80%) [11], goats in Tanzania (6.3%) [26], and sheep in Egypt (10.2%) [3], among others. Of note, there were a variety and abundance of domestic animals, wildlife (e.g., the Indian grey mongoose, *Urva edwardsii*), and humans sharing habitats in the surveyed area; therefore, cross-species (including human) transmission of zoonotic parasites is likely to occur via direct contact with animal manure or indirectly through ingestion of contaminated food or water. However, assessing the extent and frequency of such events is beyond the scope of the present study since no molecular analyses were conducted to investigate the frequency and diversity of GIP species/genotypes including the members of the *Entamoeba* complex.

The second most common protozoa found in the current study were *Eimeria* spp., with the highest rate of infection (78.3%) observed in goats followed by sheep (52%), cattle (13.6%), and camels (13.3%). Previous studies on the epidemiology of gastrointestinal protozoa in livestock have also identified *Eimeria* spp. as the most important protozoa in small ruminants in other area of Iran [27]. Likewise, in a study conducted in Yazd, Central Iran, 9.5% of slaughtered, apparently healthy, camels were positive for eimerian oocysts (*E. cameli*, *E. dromedarii*, and *E. bactriani*) at microscopy examination [28]. Furthermore, the distribution of *Eimeria* has also been investigated in one-humped camels from Mashhad in Northeastern Iran, where 18.6% of dromedaries examined were found to contain *Eimeria* spp. oocysts [29]. In a similar study conducted on fecal samples (*n* = 125) of one- and two-humped camels in Miandoab, Northwestern Iran, a 12.8% prevalence rate of coccidiosis by five *Eimeria* species (*E. bactriani*, *E. cameli*, *E. dromedarii*, *E. pellerdyi*, and *E. rajasthani*) was found [30]. Only a previous report, conducted in Zabol, was available from Southeastern Iran. In that survey, and in contrast to our study result, a high prevalence of coccidiosis (63.2%) was found among 196 asymptomatic cattle [31]. In that study, eight species of *Eimeria*, including *E. bovis*, *E. brasiliensis*, *E. cylindrica*, *E. ellipsoidalis*, *E. pellita*, *E. subspherica*, *E. wyomingensis*, and *E. zuernii,* were identified circulating in the surveyed cattle population. The fact that different species of *Eimeria* have been found in a range of diverse settings has been attributed to intrinsic differences in geographical distributions, host factors, and climatic conditions that might influence the successful sporulation of oocysts in the environment. However, it should not be overlooked that the finding of rabbit-adapted *E. pellerdyi* in camels could probably be associated with misdiagnosis.

Our findings further showed that the prevalence of *G. duodenalis* was 4.0% and 4.3% in sheep and goats, respectively. These results are consistent with many previous studies in Iran and elsewhere [2,32,33,34,35,36,37]. In two previous Iranian studies, the prevalence of *G. duodenalis* was estimated at 6.2% (12/192) in sheep, and at 5.0% (5/100) in goats in Yazd [29]. Similar results were reported in sheep (7.0%, 7/100) and goats (4.0%, 4/100) in Shiraz [6]. These prevalence rates were lower than those (14.3–42.2%) previously reported in ovine and caprine hosts in studies conducted in Iran [38], Turkey [39], and India [40]. These discrepancies in infection rates can be due to differences in animal management practices. For instance, stocking density and water supply could have an important effect on the exposure of livestock to food- and waterborne intestinal parasites, such as *G. duodenalis* [41]. The identification of *G. duodenalis* assemblages is potentially very important when evaluating the role of livestock as potential source of human giardiasis [42]. The ‘hoofed livestock’-adapted assemblage E is the most common genetic variant of *G. duodenalis* found in livestock globally, although sporadic infections by this assemblage have also been detected in humans [42]. Unfortunately, lack of molecular analyses precluded us to ascertain the genetic diversity and zoonotic potential of the *G. duodenalis* infections identified here.

In the current report, the GI nematodes were the most prevalent helminths in the examined animals. The most common type of nematode eggs found in ruminants were related to trichostrongyles, followed by *Trichuris* spp. These results were comparable to previous reports in Iran [27,43,44] and elsewhere across the globe [25,45]. Several species of the genus *Trichostrongylus*, including *T. colubriformis*, *T. vitrinus*, and *T. orientalis,* are the most important zoonotic nematode parasites, ubiquitous among herbivorous animals worldwide [46]. It is also interesting to note that most of the small ruminants that had helminths infection did not harbor concomitant obligate intracellular coccidian parasites. This result disagrees with earlier studies reporting that most of the helminth-infected small ruminants also harbored coccidian infections [47,48]. Although it was not possible to test the role of interspecific parasite interactions in influencing parasite dynamics and shaping parasite communities in this study, immunosuppression and competition have been proposed as possible explanations for the competitive presence of these parasites [49]. The most common type of nematode eggs found in donkeys were associated with strongyles. This result agrees with previous studies in Iran [4,50] and abroad [51,52].

Our investigation showed that the ruminant tapeworm *M. expansa* was found in 3.3% of camels examined. Similar infections rates in the range of 0.1–6.0% have been reported in previous studies conducted in different geographical areas, including Mashhad, Iran (4.0%, 2/50), Yazd, Iran (4.2%, 6/144), Kerman, Iran (5.0%, 3/60), Punjab, Pakistan (3.0%, 6/200), and Kassala, Sudan (0.1%, 2/1396) [20,53,54,55,56].

The present study revealed that the prevalence of amphistomes ‘rumen or stomach flukes’ in cattle was 2.3%. This result was in the lower range of those documented in previous international studies in which amphistomes were reported at prevalence rates of 8.9% in Turkey [57], of 12.1% in Algeria [58], and of 18.8% in Spain [59]. Our infection rate was also lower that those reported in other Iranian surveys conducted in Guilan (19.7%) and Mazandaran (33.9%) provinces in Northern Iran [60,61], and in Zabol province (34.6–36.9%) in Southeastern Iran [62,63].

Liver flukes of the genus *Fasciola hepatica* and *Dicrocoelium dendriticum* did not score any infection in all examined animals. This agrees with previous reports on ovine, bovine, and caprine fasciolosis in Iran [64,65] and other countries, such as Egypt [3]. However, *F. hepatica* has been found at variable prevalence rates in ruminant populations from other Iranian regions, including the Mazandaran province in Northern Iran (sheep: 7.3%; cattle: 25.4%) [66], Isfahan province in Central Iran (sheep: 3.3%; goats: 2.8%; cattle: 3.7%) [67], and the Ilam province in Western Iran (sheep: 19.0%; goats: 11.5%; cattle: 17.8%; camel: 34.6%) [68]. It should be noted that discrepant results on the prevalence rates of trematode infections in different animal populations in different studies can be attributed to variation in the climatic and ecological conditions, such as altitude, rainfall, season, temperature, sources, and types of animals involved, the response of different host species against this parasite, as well as the livestock management system among the study areas. In this regard, the absence of the digenean trematodes *F. hepatica* and *D. dendriticum* in our study area may also be attributed to the lack the suitable intermediate host species required in the life cycle of these parasites. This is the case of the amphibian snail *Galba truncatula* (the first intermediate host of *F. hepatica*) and land snails of the genera *Zebrina*, *Helicella,* and *Cionella*, and the ant species *Formica fusca*, as first and second intermediate hosts of *D. dendriticum*, respectively, in the surveyed area.

In the current study, *Taenia* spp. eggs were the only parasites found in 100% of stray dogs examined, although this result should be interpreted with caution due to the limited number of canine samples examined. These results are in line with previously published reports in Iran, in which *Taenia* spp. have been reported to be the most common tapeworms of stray dogs in Iran [16]. *Taenia hydatigena* and *T. multiceps* are two common parasites that parasitize the small intestine of dogs with their larvae causing cysticercosis tenuicollis and coenurosis in intermediate hosts, respectively [69]. Although *T. hydatigena* is not regarded as a public health concern, infection with *T. multiceps* has resulted in serious medical complications in different parts of the world due to its zoonotic potential [16].

In this study, lambs were 3.5 times more likely to be infected by coccidian parasites than adult sheep, indicating that age is one of the main factors influencing the occurrence of protozoasis in these ruminants (*p* = 0.037). In accordance with the result of the present study, other surveys conducted in Brazil, Ethiopia, Iran, and Nigeria have also reported a significantly higher prevalence of coccidiosis in lambs compared to adult sheep (*p* < 0.05) [70,71,72,73]. The high susceptibility of young animals is related to immunological aspects, with adults acquiring specific immunity against protozoa after initial exposure [74]. For instance, eimeriosis has a progressive increase in the prevalence and intensity of oocyst shedding until it reaches a peak close to the weaning period and is reduced in adults [75]. We observed that adult sheep were at higher odds of having a helminthiasis (4.1 times) when compared with lambs (*p* = 0.019). Consistent with our result, previous studies conducted among sheep and goats in Nigeria [70], Kenya [76], Ethiopia [77], and Egypt [78] have also reported the highest prevalence of helminthiasis in young animals less than one-year-old (*p* < 0.05). In contrast, a previous study conducted in Pakistan [79] reported strong associations between helminthiasis and age group <1 year in host species of buffaloes, camels, cattle, goats, and sheep (*p* < 0.05). Likewise, another study conducted in Pakistan [1] found that the overall prevalence of helminthiasis was higher in young animals compared with adults in buffaloes (*p* < 0.0001), cattle (*p* < 0.0001), goats (*p* = 0.010), and sheep (*p* = 0.059). Generally, older animals develop resistance to some of the species of the gastrointestinal nematodes; thus, young animals <2 years of age are most susceptible to infection, and it might be attributed to the immunological aspects, but it should be noted that protective immunity is a general rule and there are exceptions. In this regard, ostertagiasis, a parasitic gastritis of ruminants caused by the trichostrongyloid nematode *Ostertagia ostertagi*, has two clinical types with marked differences in features. Symptomatic type 1 occurs in young animals <2 years old during their first grazing season between July and October and it is associated with ingestion of large numbers of infective larvae. Asymptomatic type 2 occurs in adult animals >2 years old grazing on pasture (from October), and it is associated with development of hypobiotic larvae [80].

Findings reported here should be considered in the context of their strengths and limitations. In this study, the fecal samples were examined by microscopic method for the diagnosis of GIPs in domestic mammalian hosts. Because of limited diagnostic sensitivity, it is likely that prevalence rates reported here represent an underestimation of the true ones. Lack of using molecular tools to differentiate the genotypes of heterogeneous parasites, such as *Entamoeba* spp. and *G. duodenalis*, precluded us to ascertain transmission pathways or the zoonotic potential of these pathogens in the surveyed area. The small sample size for each animal is another limitation of the study. The main strength of this study is its novelty in Southeastern Iran; to the best of the author’s knowledge, this is the first study on the prevalence of GIPs among domestic mammalian hosts. The design of the study with a diverse range of animals provides a general background regarding the prevalence of parasites of these animals in the study area, which can be the basis for further hypotheses for future studies.

## 5. Conclusions

This is the first study on the prevalence of GIPs among domestic mammals in Southeastern Iran. The present study indicated age as important factor that influence risk of GIP infection in sheep in Iranshahr. This difference needs to be taken into consideration while designing control and prophylactic measures for GIP infection of sheep unique to this climatic zone and other parts of the world with similar environmental and husbandry production systems. The finding that domestic mammals harbor a similar diversity of parasites in comparison to their counterparts in wildlife suggests that regular monitoring of parasites in domestic animals is of great importance in the perspective of zoonoses. Parasitological monitoring, including testing during the rearing of free-range animals, is needed in livestock farming to detect carriers and shedders of parasite eggs, cysts, and oocysts. It is also recommended that villagers prevent stray dogs from entering agricultural fields and ensure the proper housing and management of animal facilities, with special attention to their hygienic conditions. Furthermore, accurate diagnosis of parasitic infections, as well as effective monitoring and prophylaxis, are essential for keeping livestock herds free from parasites.

## Figures and Tables

**Table 1:** Frequency distribution of GIP according to host species, age group, and sex of the animals surveyed, Iranshahr (Iran) 2022. For practical purposes, all helminthic species detected were presented together.

		*Giardia duodenalis*		*Entamoeba* spp.		*Eimeria* spp.		Helminths	
Host	Variable	Pos./Total	%	Pos./Total	%	Pos./Total	%	Pos./Total	%
Cattle	≤1 yr.	0/33	0.0	23/33	69.7	5/33	15.2	7/33	21.2
	>1 yr.	0/55	0.0	41/55	74.5	7/55	12.7	10/55	18.2
	Male	0/21	0.0	13/21	61.9	7/21	33.3	5/21	23.8
	Female	0/67	0.0	51//67	76.1	5/67	7.5	12/67	17.9
	Sub-total	0/88	0.0	64/88	72.8	12/88	13.6 ^1^	17/88	19.3 ^2^
Sheep	≤1 yr.	1/24	4.2	1/24	4.2	17/24	70.8	6/24	25.0
	>1 yr.	1/26	3.8	2/26	7.7	9/26	34.6	15/26	57.7
	Male	2/17	11.7	0/17	0.0	11/17	64.7	5/17	29.4
	Female	0/33	0.0	3/33	9.1	15/33	45.4	16/33	48.4
	Sub-total	2/50	4.0	3/50	6.0	26/50	52.0 ^3^	21/50	42.0 ^4^
Goat	≤1 yr.	1/12	8.3	1/12	8.3	9/12	75.0	2/12	16.6
	>1 yr.	0/11	0.0	0/11	0.0	9/11	81.8	5/11	45.5
	Male	1/8	12.5	1/8	12.5	7/8	87.5	2/8	25.0
	Female	0/15	0.0	0/15	0.0	11/15	73.3	5/15	33.3
	Sub-total	1/23	4.3	1/23	4.3	18/23	78.2 ^5^	7/23	30.4 ^6^
Camel	≤1 yr.	0/5	0.0	0/5	0.0	2/5	40.0	1/5	20.0
	>1 yr.	0/25	0.0	0/25	0.0	2/25	8.0	10/25	40.0
	Male	0/7	0.0	0/7	0.0	1/7	14.3	3/7	42.8
	Female	0/23	0.0	0/23	0.0	3/23	13.0	8/23	34.8
	Sub-total	0/30	0.0	0/30	0.0	4/30	13.3 ^7^	11/30	36.6 ^8^
Donkey	≤1 yr.	0/1	0.0	0/1	0.0	0/1	0.0	1/1	100
	>1 yr.	0/4	0.0	0/4	0.0	0/4	0.0	3/4	75.0
	Male	0/1	0.0	0/1	0.0	0/1	0.0	1/1	100
	Female	0/4	0.0	0/4	0.0	0/4	0.0	3/4	75.0
	Sub-total	0/5	0.0	0/5	0.0	0/5	0.0	4/5	80.0 ^9^
Dog	≤1 yr.	0/1	0.0	0/1	0.0	0/1	0.0	1/1	100
	>1 yr.	0/2	0.0	0/2	0.0	0/2	0.0	2/2	100
	Male	0/2	0.0	0/2	0.0	0/2	0.0	2/2	100
	Female	0/0	0.0	0/0	0.0	0/0	0.0	1/1	100
	Sub-total	0/3	0.0	0/3	0.0	0/3	0.0	3/3	100 ^10^

^1^ *Eimeria ellipsoidalis* (*n* = 3), *Eimeria bukidnonensis* (*n* = 1), *Eimeria auburnensis* (*n* = 8). ^2^ *Trichostrongyles* spp. (*n* = 14), *Trichuris* spp. (*n* = 1), amphistomes (*n* = 2). ^3^ *Eimeria ahsata* (*n* = 15), *Eimeria crandallis* (*n* = 10), *Eimeria intricata* (*n* = 1). ^4^ *Trichostrongyles* spp. (*n* = 18), *Trichuris* spp. (*n* = 3). ^5^ *Eimeria arloingi* (*n* = 10), *Eimeria ninakohlyakimovae* (*n* = 6), *Eimeria christenseni* (n = 3). ^6^ *Trichostrongyles* spp. (*n* = 4), *Trichuris* spp. (*n* = 3). ^7^ *Eimeria cameli* (*n* = 3), *Eimeria dromedarii* (*n* = 1). ^8^ *Trichostrongyles* spp. (*n* = 7), *Trichuris* spp. (*n* = 3), *Moniezia expansa* (*n* = 1). ^9^ *Strongyles* spp. (*n* = 4). ^10^ *Taenia* spp. (*n* = 3).

**Table 2:** Prevalence of GIP species among domestic animals in Iranshahr. 95% confidence intervals (95% CI) are indicated between brackets.

	Cattle (*n* = 88)	Sheep (*n* = 50)	Goat (*n* = 23)	Camel (*n* = 30)	Donkey (*n* = 5)	Dog (*n* = 3)
Protozoa						
*Eimeria* spp.	13.6 (7.2–22.6)	52.0 (37.4–66.3)	78.2 (56.3–92.5)	13.3 (3.7–30.7)	0 (0.0)	0 (0.0)
*Entamoeba* spp.	72.8 (62.2–81.7)	6.0 (1.2–16.5)	4.3 (0.1–21.9)	0 (0.0)	0 (0.0)	0 (0.0)
*Giardia duodenalis*	0 (0.0)	4.0 (0.5–13.7)	4.3 (0.1–21.9)	0 (0.0)	0 (0.0)	0 (0.0)
Helminths						
Amphistomes	2.3 (0.2–7.9)	0 (0.0)	0 (0.0)	0 (0.0)	0 (0.0)	0 (0.0)
*Moniezia expansa*	0 (0.0)	0 (0.0)	0 (0.0)	3.3 (0.1–17.2)	0 (0.0)	0 (0.0)
Strongyles spp.	0 (0.0)	0 (0.0)	0 (0.0)	0 (0.0)	80 (28.3–99.4)	0 (0.0)
*Taenia* spp.	0 (0.0)	0 (0.0)	0 (0.0)	0 (0.0)	0 (0.0)	100 (29.2–100)
Trichostrongyles spp.	15.9 (8.9–25.2)	36 (22.9–50.8)	17.3 (4.9–38.7)	23.3 (9.9–42.2)	0 (0.0)	0 (0.0)
*Trichuris* spp.	1.1 (0.1–6.1)	6.0 (1.2–16.5)	13 (2.7–33.6)	10 (2.1–26.5)	0 (0.0)	0 (0.0)

**Table 3:** Frequency distribution of GIP according to host age and sex in Iranshahr; 95% confidence intervals (95% CI) and odd ratios (OR) are indicated.

	Protozoa					Helminths				
Variable	Total (*n*)	Infected (*n*)	%	OR	95% CI	Total (*n*)	Infected (*n*)	%	OR	95% CI
Cattle (*n* = 88)										
≤1 yr.	33	26	78.8	1.0	Reference	33	7	21.2	1.2	0.34–4.02
>1 yr.	55	46	83.6	1.4	0.38–4.69	55	10	18.2	1.0	Reference
Male	21	19	90.5	2.5	0.49–24.57	21	5	23.8	1.4	0.34–5.21
Female	67	53	79.1	1.0	Reference	67	12	17.9	1.0	Reference
Sheep (*n* = 50)										
≤1 yr.	24	18	75.0	3.5	1.05–11.66	24	6	25.0	1.0	Reference
>1 yr.	26	12	46.2	1.0	Reference	26	15	57.7	4.1	1.06–16.59
Male	17	13	76.5	3.1	0.72–15.29	17	5	29.4	1.0	Reference
Female	33	17	51.5	1.0	Reference	33	16	48.4	2.3	0.56–9.96
Goat (*n* = 23)										
≤1 yr.	12	9	75.0	1.0	Reference	12	2	8.3	1.0	Reference
>1 yr.	11	9	81.8	1.5	0.13–21.71	11	5	45.5	4.2	0.45–53.54
Male	8	7	87.5	2.5	0.23–27.71	8	2	12.5	1.0	Reference
Female	15	11	73.3	1.0	Reference	15	5	33.3	1.5	0.16–20.18
Camel (*n* = 30)										
≤1 yr.	5	2	40.0	7.7	0.77–76.45	5	1	20.0	1.0	Reference
>1 yr.	25	2	8.0	1.0	Reference	25	10	40.0	2.7	0.26–27.49
Male	7	1	14.3	1.1	0.02–17.15	7	3	42.8	1.4	0.16–10.64
Female	23	3	13.0	1.0	Reference	23	8	34.8	1.0	Reference

## Data Availability

Data supporting the findings of this study are contained within the article.
